# Differential physiological and biochemical responses under variable culture conditions in micro-propagated *Solenostemon scutellarioides*: an important ornamental plant

**DOI:** 10.1007/s13659-012-0035-y

**Published:** 2012-07-07

**Authors:** Ranabir Sahu, Saikat Dewanjee

**Affiliations:** Advanced Pharmacognosy Research Laboratory, Department of Pharmaceutical Technology, Jadavpur University, Kolkata, 700032 West Bengal India

**Keywords:** anthocyanin, chlorophyll, *in vitro*, micropropagation, *Solenostemon scutellarioides*

## Abstract

*Solenostemon scutellarioides* is a commercially important ornamental plant. In present study, it was attempted to establish a protocol for high frequency *in vitro* regeneration of *S. scutellarioides*. Caulogenesis was found to be significant in solid MS medium supplemented with 0.5 mg dm^−3^ BAP. Combination of GA_3_ (0.5 mg dm^−3^) and BAP (0.5 mg dm^−3^) induced high frequency regeneration coupled with higher plant height. The plantlets exposed to IBA (1 mg dm^−3^) exhibited significant root development in terms of first appearance of root and number of roots per shoot. To improve its commercial acceptability in terms of plant architecture and foliage colouration, two weeks old *in vitro* grown plantlets were exposed to different culture conditions namely MS strength, sucrose concentration, pH and light. A variable plant types with diverse ornamental traits were developed under different culture conditions.

**Electronic Supplementary Material:**

Supplementary material is available for this article at 10.1007/s13659-012-0035-y and is accessible for authorized users.

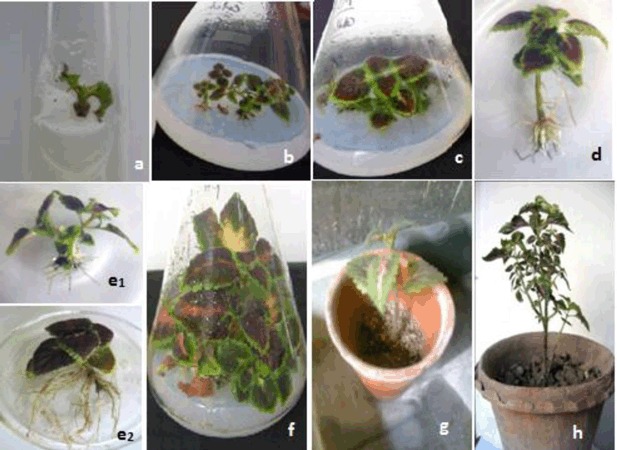

## Introduction

*Solenostemon scutellarioides* (L.) Codd (syn. *Coleus blumei* Benth.), commonly known as Coleus or Painted Nettle, is an important ornamental plant valued notably for its vibrant colourful foliage.[1] In the last decade, this ornamental plant has gained popularity in the floriculture industry with the introduction of a large number of vegetatively propagated varieties with novel foliage colours. Besides novel foliage colours, opportunities exist to increase their commercial value by manipulating plant architecture. Several hundred commercial cultivars are characterized by their leaf colors, leaf shape, plant architecture and flowering characteristics. The desirable characteristics in *S. scutellarioides* include improved plant architecture and foliage colour quality. Bright colourful foliages improve its artistic appeal[2] and faded colours decrease its product value.[3] Considering these traits, commercial *Coleus* breeding was initiated to develop new cultivars through traditional breeding methods for improved commercial variety. The commercial cultivation of *S. scutellarioides* is primarily done by cutting method to regenerate similar novel variety. However, to meet with increasing commercial demand high frequency regeneration is desirable and employment of plant tissue culture may serve as an alternative source of *S. scutellarioides* for commercial purpose.

*To whom correspondence should be addressed. E-mail: s.dewanjee@yahoo.com

Plant architecture in *S. scutellarioides* mainly includes the branching pattern of shoots and leaf morphology. Increasing branching with improved leaf morphology is the main criteria for the selection of better commercial variety. Colouration of different organs such as flower, fruit and leaf is due to the accumulation of chlorophyll, betalains, carotenoids, flavnoids, anthocyanins etc,[4] with the latter as the major pigment that impart the red and purple colours observed in *S. scutellarioides* leaves.[5] The attractive colouration of *S. scutellarioides* leaves is affected by the combination of anthocyanin and chlorophyll content, which is responsible for green colouration.[6] Foliage colour may be dependent on factors such as light and pH, analogously to what has been observed in flowers.[7,8] Efforts to manipulate plant architecture and foliage colouration to improve commercial acceptability and manipulation of culture condition would serve productive. Therefore the objective of the present work is to establish a protocol for rapid clonal multiplication from limited donor plants and developing different commercial plant types under different culture conditions. The each type with different architecture and foliage colour would serve as the source of explants and mass multiplication achieved by using rapid clonal multiplication protocol.

## Results and Discussion

Nodal segments containing axillary buds have the potential to develop complete plantlets in a nutrient medium containing suitable combinations and concentrations of growth regulators.

To optimize high frequency regeneration, surface sterilized nodal segments of *S. scutellarioides* were cultured in MS media containing 3% sucrose and pH 5.6. After one week, explants showing axillary buds were transferred into Murashige and Skoog (MS)[9] media supplemented with different combinations of phytohormones for organogenesis. Cytokinins are known to stimulate plant cell division and participate in the release of lateral bud dormancy, induction of adventitious bud formation and in growth of lateral buds.[10] Different concentrations (0.5 and 1.0 mg $dm^{-3}$) of cytokinins namely 6-benzylaminopurine (BAP) and kinetin (Kn) were used for the shoot regeneration (caulogenesis). Shoot regeneration response was expressed as number of regenerated shoots per explant and plant height (Figure 1 (a–c), 2a). No detrimental effect was noticed in terms of plant survival with cytokinin treatment. The overall effect of cytokinins on plant height was not promising, which resulted dwarf plantlets with reduced leaf size. The only positive effect was observed in number of shoot generation per explant. BAP (0.5 mg $dm^{-3}$) was observed to be most beneficial for high frequency regeneration of shoots. No significant difference was observed in terms of plant stature and foliage colour (green with brownish shade) with BAP and Kn treatment. The positive effect of BAP at low concentration was also observed in *Coleus forskohlii*[11] and *Plumbago indica.*[12]

**Figure 1.** Different stages of micropropagation of *S. scutellarioides* (a–h): (a) sprouting of shoot buds; (b) initiation of shoot regeneration; (c) emergence of multiple shoot development in BAP (0.5 mg $dm^{-3}$); (d) enhancement of shoot height with good survival rate in $GA_3$ + BAP (0.5 mg $dm^{-3}$ each); ($e_1$) initiation of roots at the base of single micro-shoot; ($e_2$) better rooting in media supplemented with IBA (1.0 mg $dm^{-3}$) at the same period of culture ($e_1$)'; (f) well established *in vitro* culture; (g) a hardened plant in glass house; (h) micropropagated plant growing under *ex vitro* environment

Gibberellic acid ($GA_3$) is best known for its role in elongation of axial organs, such as stems, petioles and inflorescences.[13] The effect of $GA_3$ on shoot elongation is due to the increase in soluble carbohydrates induced by $GA_3$ and available for metabolic processes.[14] In present study, $GA_3$ exhibited significant improvement in height especially to the plants previously treated with BAP (dwarf in nature) (Figure

**Figure 2.** (a) effect of cytokinins on shoot regeneration; (b) effect of $GA_3$ on shoot elongation; (c) effect of auxins on root regeneration from nodal explants of *S. scutellarioides*

2b). The negative effect of $GA_3$ was observed on survival percentage after one week, particularly to those plants were exposed with higher concentration of $GA_3$. Severe chlorosis was observed in the plants treated with $GA_3$ (1.0 mg $dm^{-3}$). But the deleterious effect of $GA_3$ was minimized when it was used in combination with BAP. Combination of $GA_3$ and BAP at low concentration exhibited high frequency regeneration coupled with higher plant height. Combination of $GA_3$ (0.5 mg $dm^{-3}$) and BAP (0.5 mg $dm^{-3}$) exhibited best result with almost 1.4 and 1.3 fold increase in shoot numbers and plant height respectively than control (Figure 2b). Plant stature was normal as in control but some changes in plant colour were observed. A pinkish colouration was developed on the upper surface of leaf especially near to mid vein. Leaf area was broader than control and cytokinins treated plant. Leaf margin was serrated which was not common in other treatment. The positive effect of $GA_3$ and BAP in combination was also reported in *Ocimum basilicum*.[15]

Auxins exert a strong influence in root formation.[16] For root generation (rhizogenesis), *in vitro* regenerated and elongated shoots were transferred into MS medium supplemented with different concentrations (0.5, 1.0 and 1.5 mg $dm^{-3}$) of indole-3-acetic acid (IAA) and indole-3-butyric acid (IBA). In course of culture, roots appeared from the base of shoots. The first appearance of root and number of roots per shoot were recorded (Figure 1 ($e_1$, $e_2$), 2c). Both IAA and IBA showed positive effect for early rooting as well increase number of roots per shoot. Plantlets treated with IBA (1 mg $dm^{-1}$) exhibited best result as roots appeared earliest with almost 2.3 fold increase in number of roots per shoot as compared with control culture. Auxins treatment did not cause any significant morphological changes namely plant architecture and foliage colour. The success of IBA in promoting root induction has been reported in *Ceropegia candelabrum*[17] and *Hemidesmus indicus*.[18]

To study the effect of different external stimuli, two weeks old *in vitro* grown shoots were exposed with different culture conditions. The regenerated plants maintained in different culture conditions exhibited variable morpho-chemical traits to develop different plant lines with respect to architecture and foliage colouration (Figures 3–6). To study the effect of MS strength, the growth and regeneration potential was found to be improving with MS strength from 1/4 to 1 (Figure 3) but detrimental effect was observed in the cultures treated with 2MS strength. The foliage colours were also significantly varied. The culture with 1/2 and 1 MS strength exhibited beautiful foliage pattern with attractive colouration due to better anthocyanins concentration and selected as developed plant types. The effect of pH of culture media on plant architecture and leaf pattern largely varied (Figure 4). pH did not show significant variation in regeneration potential but it imparted significant difference in leaf morphology and colouration. The plants exhibited diverse attractive foliage colouration between pH 3.6–5.6. The effect of pH on both leaf morphology and foliage colour was found to be optimum at pH 5.6. Higher pH at 7.6 was found to be detrimental for the growth of *S. scutellarioides.* The plant exposed to pH 4.6 and 5.6 showed highest chlorophyll and anthocyanin content respectively. Amongst the selection, the plants regenerated in pH 3.6, 4.6 and 5.6 were chosen as developed elite types. The effect of sucrose concentration in MS media on plant architecture and foliage pattern was studied (Figure 5). Sucrose concentration did not have significant effect in shoot regeneration per explant but the height improvement was found to be optimum in culture treated with 3% sucrose. Foliage morphology and colouration was found to better in culture treated with 3% sucrose. Chlorophyll concentration in leaves gradually decreased with increasing sucrose concentration. Though sucrose concentrations gave diverse foliage colouration but for the selection of plant type, plants treated with 3% and 4% sucrose were chosen due to better leaf surface and healthy leaf structure. The effect of light on growth and regeneration potential of *S. scutellarioides* was gradually decreased from white, yellow, green, blue and black exposure (Figure 6). Leaf morphology remained almost similar in white, yellow and green light treatment. Detrimental effect was observed in absence of light. It also significantly reduced ornamental aspect of *S. scutellarioides* leaves. Chlorophyll and anthocyanin concentrations also largely varied. Green light improved chlorophyll content while white light showed higher anthocyanin content in leaves. Exposure with yellow light exhibited an intermediate concentration of both pigments. Exposure with blue light significantly reduced anthocyanins content.

The high diversity of colour in foliage of *S. scutellarioides* has been shown to be due variation of anthocyanins and

**Figure 3.** Effect of MS strengths on plant architecture and foliage colour development of *S. scutellarioides*

chlorophyll ratio. Anthocyanins are key pigment for attractive pink to purple colouration in *S. scutellarioides.* Anthocyanins are water soluble pigment having high ecophysiological significance in plant.[19] Several hypotheses described that the role of anthocyanins in osmotic regulation to drought and frost stress, antioxidant defense and protection from different light exposure. Based on the hypothesis to develop of new varieties of *S. scutellarioides* with improved colour quality, the shoots *in vitro* derived were exposed *in vitro* with various factors namely MS strength, sucrose concentration, pH and light. In this study, it was observed that selected factors had significant

**Figure 4.** Effect of pH on plant architecture and foliage colour development of *S. scutellarioides*

impact on foliage colour development and leaf architecture during developmental stage and the develop plants carried these traits up to *ex vitro* establishment. Therefore the developed variable plant types with diverse ornamental traits would serve as a secondary source of *S. scutellarioides* for commercial purpose.

## Experimental Section

**Collection of Plant Material.** *S. scutellarioides* L. plants were collected from Medicinal Plant Garden of Narendrapur Ramkrishna Mission, South 24 Parganas, India. The plant was authenticated by the Taxonomists of Botanical Survey of India, Shibpur, India. A voucher specimen JU/PT/PC/01/06 was

**Figure 5.** Effect of sucrose concentrations on plant architecture and foliage colour development of *S. scutellarioides*

deposited at our laboratory for future reference. The plants were planted in the medicinal plant garden at the Department of Pharmaceutical Technology, Jadavpur University, India.

**Preparation and Sterilization of Explants.** Nodal segments (0.4 ± 0.1 cm) with internodes were excised from young branches of the plants. The explants containing two buds with oppositely placed axils were used for the experiment. The explants were first washed with running tap water for removing adhering dust particles and then immersed in 1% Teepol

**Figure 6.** Effect of light on plant architecture and foliage colour development of *S. scutellarioides*

detergent (Glaxo India Pvt. Limited, India) for 5 min with continuous shaking in a magnetic stirrer. After gentle washing with sterile distilled water for 4-5 times, the clean segments were surface sterilized with 0.1% mercuric chloride for 1 min, followed by 3-4 times washing with autoclaved distilled water.

***In Vitro* Establishment.** After surface sterilization, each sterile explant was vertically implanted into single culture tube (25 × 150 mm, Borosil, India) contained 25 $cm^3$ sterilized solid MS media. The media was fortified with 3% sucrose (Hi Media, India) gelled with 0.8% Bacto agar (Hi-media, India). The pH was adjusted to 5.6 before autoclaving at 121 ºC for 20 minutes. All cultures were maintained at 22 ± 2 °C and 16 h photoperiod (light intensity 100 $\mu$mol $m^{-2}$ $s^{-1}$ provided by cool white fluorescent tubes, Philips, India). For adventitious bud induction, 1 week old explants were sub-cultured into 250 $cm^3$ conical flasks containing 50 $cm^3$ solid MS medium supplemented with different concentrations of two cytokinins (BAP and Kn). After two weeks, the conical flasks exhibited significant caulogenesis were sub-cultured in media containing $GA_3$ singly or in combination with BAP at different concentrations for better shoot development. To study the effect of auxins on rhizogenesis, the healthy *in vitro* grown plants were transferred in solid MS medium supplemented with IAA and IBA at different concentrations. The best resulting media for rooting was identified on the basis of first appearance of root and number of roots per shoot on day 14.

***Ex Vitro* Establishment.** After two weeks, healthy plantlets with sufficient roots were removed from *in vitro* condition for hardening. The adhered agar was removed by repeated washing with autoclaved distilled water and the plantlets were transferred to small pots (1 for 1 plantlet), filled with autoclaved soilrite and covered with transparent polythene bags under tissue culture environment. After one week, the plantlets resumed growth were again hardened with autoclaved soilrite-soil mixture (1:1) under same condition for another week. The well hardened plants were transferred to glass house in the large earthen pots for full growth of plants under normal environmental condition for the acclimatization. After two weeks the survived plants were finally transferred in the field.

**Manipulation of Media and Culture Conditions to Manipulate Plant Architecture and Foliage Color.** To study the effect of different culture conditions two weeks old *in vitro* grown shoots were exposed with various factors namely MS strength, sucrose concentration, pH and light. One set of culture in MS with 3% sucrose, pH 5.6, under white light was maintained throughout experiment as control. Data were recorded as number of shoots per explant, plant height, leaf surface and foliage chlorophyll and anthocyanin content.

Total anthocyanins were extracted as previously described[20] and absorbance was determined by spectrophotometry at 535 nm. Anthocyanin content was calculated according to the method of Murray and Hackett[21] with little modification.[4]

For chlorophyll determination, collected leaves were frozen in liquid nitrogen. The samples were ground and chlorophyll was extracted. The absorbance was determined at 649 nm and 665 nm. The total chlorophyll a/b concentration was calculated as previously described.[22] The *ex vitro* establishment of different plant lines were done as similar described in previous section.

**Statistical Analysis.** Each set of experiment consisted of 10 replicates and represented by 10 individual conical flasks. Each experiment was repeated thrice and the data were analyzed statistically using SPSS software (10.0.5, 1999, SPSS Inc).Variability in data was expressed as the mean ± standard deviation.

**Electronic Supplementary Material**

Supplementary material is available in the online version of this article at http://dx.doi.org/ 10.1007/s13659-012-0035-y and is accessible for authorized users.

**Acknowledgments**

The authors are thankful to University Grants Commission, New Delhi, India for providing fund to Mr Ranabir Sahu through Rajiv Gandhi National Fellowship, 2011. We are also thankful to Dr. Md. Nasim Ali, Ramkrishna Mission Vivekananda University, Ramkrishna Mission Ashrama Narendrapur, India for providing plant material, *S. scutellarioides,* for this study.

**Open Access** This article is distributed under the terms of the Creative Commons Attribution License which permits any use, distribution, and reproduction in any medium, provided the original author(s) and source are credited.

## Electronic supplementary material

Supplementary material, approximately 127 KB.
