# Supplementary material for: Differential physiological and biochemical responses under variable culture conditions in micro-propagated Solenostemon scutellarioides: an important ornamental plant
Source: Nat Prod Bioprospect. 2012 Jul 7;2(4):160–5. doi: 10.1007/s13659-012-0035-y (PMC4131626; doi:10.1007/s13659-012-0035-y)
Supplement: Supplementary file 1 — Supplementary material, approximately 127 KB. [file 13659_2012_35_MOESM1_ESM.pdf]

Electronic Supplementary Material

Differential physiological and biochemical responses under variable culture conditions in micro-propagated *Solenostemon scutellarioides*: an important ornamental plant

Ranabir SAHU, and Saikat DEWANJEE*

Advanced Pharmacognosy Research Laboratory, Department of Pharmaceutical Technology, Jadavpur University, Kolkata 700032, West Bengal, India

Received 11 May 2012; Accepted 28 June 2012

Abstract: *Solenostemon scutellarioides* is a commercially important ornamental plant. In present study, it was attempted to establish a protocol for high frequency *in vitro* regeneration of *S. scutellarioides.* Caulogenesis was found to be significant in solid MS medium supplemented with 0.5 mg dm^{-3} BAP. Combination of GA_3 (0.5 mg dm^{-3}) and BAP (0.5 mg dm^{-3}) induced high frequency regeneration coupled with higher plant height. The plantlets exposed to IBA (1 mg dm^{-3}) exhibited significant root development in terms of first appearance of root and number of roots per shoot. To improve its commercial acceptability in terms of plant architecture and foliage colouration, two weeks old *in vitro* grown plantlets were exposed to different culture conditions namely MS strength, sucrose concentration, pH and light. A variable plant types with diverse ornamental traits were developed under different culture conditions.

*To whom correspondence should be addressed. E-mail: s.dewanjee@yahoo.com

Numerical data expressing the effect of cytokinins on shoot regeneration

	Number of shoots/explant	Plant height
Control	4.77±0.74	4.93±0.5
BAP (0.5 mg dm-3)	6.73±1.12	4.23±0.25
BAP (1.0 mg dm-3)	5.97±1.46	4.03±0.21
Kn (0.5 mg dm-3)	5.40±0.57	4.13±0.45
Kn (1.0 mg dm-3)	5.03±0.65	3.87±0.42

Numerical data expressing the effect of GA_3 and in combination with BAP on shoot elongation

	No of shoots /explant	Plant height
Control	4.7± 0.72	6.12±0.62
GA3 (0.5 mg dm-3)	4.76±0.81	8.06±0.79
GA3 (0.5 mg dm-3) + BAP (0.5 mg dm-3)	6.37±1.10	8.23±0.74
GA3 (0.5 mg dm-3) + BAP (1.0 mg dm-3)	5.73±1.33	7.47±0.95
GA3 (1.0 mg dm-3)	4.73±0.75	7.8±0.86
GA3 (1.0 mg dm-3) + BAP (0.5 mg dm-3)	5.17±0.65	7.13±0.42
GA3 (1.0 mg dm-3) + BAP (1.0 mg dm-3)	4.87±0.50	7.01±0.45

Numerical data expressing the effect of auxins on root regeneration

	First appearance of root (day)	Number of roots/shoot
Control	8.19±0.56	14.17±4.58
IAA (0.5 mg dm-3)	7.51±0.78	23.67±3.98
IAA (1.0 mg dm-3)	6.91±0.87	23.83±6.65
IAA (1.5 mg dm-3)	7.67±0.67	22.5±5.34
IBA (0.5 mg dm-3)	7.42±0.68	28.67±3.56
IBA (1.0 mg dm-3)	6.67±0.45	32.33±3.24
IBA (1.5 mg dm-3)	7.22±0.76	29.33±4.34

Numerical data expressing the effect of pH, MS, sucrose and light on ornamental aspects

Parameters	No of shoots/explant	Plant height (cm)	Leaf area (cm^2)	Chlorophyll (649 nm + 665 nm)	Anthocyanins (535 nm)
1/4 MS	3.45±0.45	3.67±0.26	2.34±0.38	11.8	0.28
1/2 MS	4.12±0.32	4.85±0.22	4.01±0.32	9.6	0.48
MS	4.98±0.36	6.28±0.56	4.10±0.62	10.8	0.52
2 MS	3.45±0.28	3.12±0.35	2.18±0.22	9.8	0.3
pH 3.6	4.08±0.65	3.98±0.38	2.88±0.24	9.5	0.45
pH 4.6	4.12±0.42	4.62±0.32	2.89±0.28	13.7	0.42
pH 5.6	4.98±0.36	6.28±0.56	4.10±0.62	10.8	0.52
pH 7.6	3.48±0.38	3.12±0.24	2.82±0.32	9.2	0.24
Sucrose (1%)	4.20±0.45	3.27±0.18	2.22±0.27	13.8	0.32
Sucrose (2%)	4.81±0.52	4.01±0.24	2.25±0.18	12.7	0.42
Sucrose (3%)	4.98±0.36	6.28±0.56	4.10±0.62	10.8	0.52
Sucrose (4%)	3.9±0.28	5.82±0.34	2.84±0.22	9.8	0.4
White	4.98±0.36	6.28±0.56	4.10±0.62	10.8	0.52
Yellow	4.34±0.32	5.67±0.22	3.90±0.51	13.5	0.42
Green	3.76±0.28	4.24±0.25	3.92±0.42	14.2	0.34
Blue	3.45±0.28	5.12±0.35	3.16±0.45	13.2	0.24
Black	2.34±0.22	2.94±0.31	1.98±0.18	8.12	0.1
